# Emergence of West Nile Virus Lineage 2 in Europe: A Review on the Introduction and Spread of a Mosquito-Borne Disease

**DOI:** 10.3389/fpubh.2014.00271

**Published:** 2014-12-08

**Authors:** Luis M. Hernández-Triana, Claire L. Jeffries, Karen L. Mansfield, George Carnell, Anthony R. Fooks, Nicholas Johnson

**Affiliations:** ^1^Wildlife Zoonoses and Vector-Borne Diseases Research Group, Animal and Plant Health Agency, Addlestone, UK; ^2^London School of Hygiene and Tropical Medicine, London, UK; ^3^Department of Clinical Infection, University of Liverpool, Liverpool, UK

**Keywords:** West Nile virus, lineage, emergence, encephalitis, Europe

## Abstract

West Nile virus (WNV) is transmitted by mosquitoes and causes fever and encephalitis in humans, equines, and occasionally wild birds. The virus was first isolated in sub-Saharan Africa where it is endemic. WNV lineage 1 has been responsible for repeated disease outbreaks in the countries of the Mediterranean basin over the past 50 years. This lineage was also introduced into North America in 1999 causing widespread human, equine, and avian mortality. WNV lineage 2, the first WNV lineage to be isolated, was believed to be restricted to sub-Saharan Africa causing a relatively mild fever in humans. However, in 2004, an investigation in Hungary of a case of encephalitis in a wild goshawk (*Accipiter gentiles*) resulted in the isolation of WNV lineage 2. During the summer of 2004, and in subsequent years, the virus appeared to spread locally throughout Hungary and into neighboring Austria. Subsequently, WNV lineage 2 emerged in Greece in 2010 and in Italy in 2011, involving outbreaks on the Italian mainland and Sardinia. Further spread through the Balkan countries is also suspected. Whole genome sequencing has confirmed that the virus responsible for the outbreaks in Greece and Italy was almost identical to that isolated in Hungary. However, unlike the outbreaks in Hungary, the burden of disease in Mediterranean countries has fallen upon the human population with numerous cases of West Nile fever and a relatively higher mortality rate than in previous outbreaks. The emergence of WNV lineage 2 in Europe, its over-wintering and subsequent spread over large distances illustrates the repeated threat of emerging mosquito-borne diseases. This article will review the emergence of WNV lineage 2 in Europe; consider the pathways for virus spread and the public health implications for the continent.

## Introduction

In recent years, arthropod-borne viruses have shown an increasing ability to spread beyond the areas, which had been considered to be their established geographic ranges. A number of factors are driving this process including bird migration, increasing global trade, and the movement of vector species (1). This range expansion threatens public and livestock health. Examples of viruses that have emerged in Europe and which are pathogenic for livestock include bluetongue virus (2) and most recently Schmallenberg virus (3). Such disease outbreaks incur both economic and animal health costs that threaten the livestock industry. Other emerging viruses are zoonotic, and the repeated emergence of West Nile virus (WNV) in Europe is a particular example of one such range expansion (4). The ability to detect and respond to emerging disease outbreaks, through rapid pathogen testing and host-specific serological assays, is a key component for disease response. This review considers the emergence of WNV lineage 2 in Europe as an example of the threat to public and livestock health from emerging zoonoses.

West Nile virus is classified within the genus *Flavivirus*, family *Flaviviridae*, and is phylogenetically and antigenically related to Japanese encephalitis virus. The virus was first isolated from the blood of a woman suffering a febrile illness in the West Nile district of Uganda (5). This first isolate is now believed to belong to lineage 2 suggesting its early zoonotic potential. Subsequent studies made further isolations of WNV from human sera in Egypt (6), and from birds and mosquitoes (7). This established that mosquitoes were the likely virus vector and through blood-feeding on birds, the virus was maintained in an endemic cycle. Early phylogenetic studies (discussed in more detail in subsequent sections) demonstrated that there are two major lineages, both present in Africa (8). Subsequent events including the emergence of WNV in North America, its spread throughout the western hemisphere, and repeated outbreaks in Europe suggest that WNV has the largest distribution of any arthropod-borne virus. In Africa, WNV is endemic and widely distributed. Human cases have been sporadic, but environmental conditions favoring mosquitoes, such as high diurnal temperatures or frequent rainfall, have led to large epidemics (9). One such episode in the Karoo region of South Africa in 1974 involved tens of thousands of human infections. Currently, approximately 5–15 human cases are confirmed each year in South Africa, however, only a small proportion of cases undergo laboratory investigation, so this may be a considerable underestimate of the actual number of infections (9). In horses and birds, serology studies have demonstrated a high seroprevalence of WNV infection in southern Africa (10, 11).

Annual late-summer outbreaks of WNV are now a regular occurrence in European countries that border the Mediterranean Sea and the virus is now considered endemic in some regions (12, 13). Most outbreaks have been identified as WNV lineage 1 and were closely related to outbreaks in Israel and North America (14). This lineage has also been responsible for deaths in humans, horses, and avian species. Strikingly, in Africa, where lineage 2 predominates, relatively few cases of neurological disease are reported, whereas in North America and Europe, numerous human and equine cases have occurred, leading to the suggestion that lineage 1 strains had increased pathogenicity, while lineage 2 strains were of low virulence (9). However, experimental studies in mice (15) and case reports (16, 17), have demonstrated that both WNV lineages have the ability to cause zoonotic disease, with the potential for fatal neuroinvasive disease. This has been realized fully with repeated outbreaks of West Nile fever in Greece since 2010, caused by WNV lineage 2, resulting in hundreds of human cases of West Nile neurological disease (WNND) (18).

## Emergence of WNV Lineage 2 in Europe

The first WNV isolated in 1937 in Uganda has since been shown to be a lineage 2 isolate, and for many years this lineage was believed to be restricted to sub-Saharan Africa (5). Until the early 2000s, WNV infections beyond Africa, including its emergence in North America, and Kunjin virus in Australia, were caused by viruses within lineage 1 (19). This included a number of outbreaks in Europe and countries around the Mediterranean Basin (12). Outbreaks of West Nile disease were recorded in Algeria (1994), Morocco and Romania (1996), Tunisia (1997), Italy (1998), Israel and Russia (1999), and France (2000). Detailed phylogenetic analysis of viruses isolated from these outbreaks suggested that those around the western Mediterranean were caused by a single strain, referred to as the WMed subtype, and that this was a single introduction of virus that overwintered over a number of years (20). It was conjectured that this sub-lineage was transferred between Mediterranean countries by viremic birds, leading to the initiation of new outbreaks. A second closely related sub-lineage included viruses isolated from Romania and Russia, and a more divergent sub-lineage was responsible for outbreaks in Israel and North America. Each sub-lineage likely represents a separate introduction of WNV into Europe from Africa.

**Figure 1:**
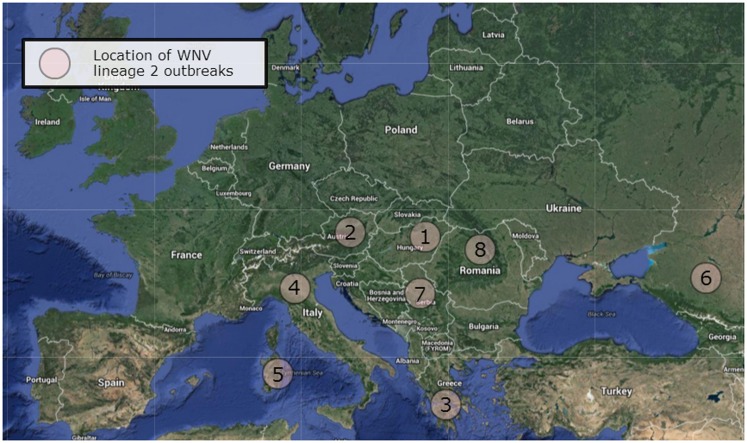
**West Nile virus lineage 2 outbreaks in Europe**. The outbreaks occurred in Hungary (1), Austria (2), Greece (3), Italy (4), Sardinia (5), Russia (6), Serbia (7), and Romania (8). Map data: Google maps.

West Nile lineage 2 was first detected in Europe in 2004 with its isolation from the brain of a goshawk (*Accipiter gentiles*) in Hungary (21). A human case of WNV lineage 2 infection was retrospectively confirmed to have occurred in Russia in the same year (22). Subsequent surveillance between 2004 and 2009 of dead birds of prey, especially in goshawks, led to repeated isolations of the virus across Hungary (23). The species nests and thrives in areas of deciduous and coniferous forests and so would be targeted by ornithophilic *Culex pipiens* complex mosquitoes, the main transmitters of WNV in Europe. The prevalence of infection in the northern goshawk could also result from oral transmission as this species preys on other birds, particularly pigeons. Local spread resulted in WNV infection of raptors in Austria (24). Subsequent outbreaks have occurred in a number of other European countries including Austria, Greece, Romania, Serbia, and Italy (Table 1; Figure 1).

**Table 1:** **Information on confirmed outbreaks of WNV lineage 2 in Europe between 2004 and 2013**.

Country	Year	Species affected	Reference
Russia	2004	Human	(22)
Hungary	2004–2008	Wild birds, sheep, horses, human	(21, 23)
Austria	2008	Wild birds	(24)
Greece	2010	Human, wild birds, mosquitoes	(25, 26)
Romania	2010	Human	
Russia	2011	Human	(22)
Italy	2011	Human, wild birds, mosquitoes	(27, 28)
Italy (Sardinia)	2012	Human	(29–31)
Serbia	2012	Human	
Italy	2013	Human	

The outbreak in Greece has been particularly severe. The virus was first detected in 2010 (32) in Northern Greece and developed, in contrast to the emergence in Hungary, into a large human epidemic. The majority of cases were reported west of the city of Thessaloniki, between the rivers Axios and Aliakmonas. Retrospective serology suggests that this virus, or a related one, had been circulating in Greece for some years prior to the first human cases of WN fever (WNF) (33). However, this was at a low level, <1% seropositivity, and was not accompanied by reports of disease. The first case in 2010 occurred in early July and incidence peaked in mid-August. The last cases occurred in early October. In total, 262 patients were recorded, with 65 classified as West Nile fever and 197 suffered neurological disease, of which 33 died (34). Age profiling demonstrated that the elderly were most at risk of disease, particularly those over 70 years of age, and risk was further increased if the individual had an existing medical condition such as hypertension, heart disease, or diabetes. Subsequent surveillance isolated WNV lineage 2 from mosquitoes (35), giving rise to the Nea Santa-Greece 2010 strain, and from wild resident birds (36). Epidemics of WNV lineage 2 have occurred during the late summer in Greece in both 2011 and 2012. Further sequencing of viruses detected in humans has confirmed that the same virus strain is present in both humans and wildlife and has been present in each subsequent year, suggesting endemic persistence in Greece (37). This strain appears to cause disease in humans and wild avian species with relatively few confirmed reports of disease in equine species.

West Nile lineage 2 emerged in Italy in 2011, the year after the first detection in Greece. The first reported case occurred in a man in his late 50s in the coastal town of Ancona on the Adriatic Sea (27). The patient reported symptoms of malaise and fever early in September, and was admitted to hospital. With the patient reporting no history of travel outside of Italy, this was considered an autochthonous case. Shortly after this, six cases of neurological disease due to WNV lineage 2 were reported in Sardinia between September and October, 2011 (28). Mosquito surveillance for WNV detected lineage 2 in *Cx. pipiens* mosquito pools and in a collared dove (*Streptopelia decaocto*) in northern Italy, where lineage 1 has been endemic since 2008 (38). Detections of WNV lineage 2 in Italy have continued in subsequent years, and have included further human cases (29, 31). As in Greece, instances of WNV infection in horses in Italy are rare.

West Nile fever was detected in humans in Romania in 2010 and Serbia in 2012. In Romania 57 cases were reported, 54 with neuroinvasive disease (26). In Serbia, 58 patients were confirmed infected (30). Of these, 52 developed neuroinvasive disease and 9 died. Virus isolation and phylogenetic analysis confirmed that both outbreaks were due to WNV lineage 2 (26, 39).

There is currently no vaccine against WNV licensed for human use, and although WNV lineage 2 has not been detected in countries in northern Europe, travelers to affected areas during the periods of vector activity are at risk of infection. This was illustrated by a human case of WNV infection in a 73 years-old Belgian woman who was visiting Greece in the summer of 2012 (40). Following her return to Belgium, samples of serum and cerebrospinal fluid were both positive for WNV IgM and IgG. The serum sample, taken 29 days after development of fever, was positive for WNV by real-time RT-PCR. A 116 base pair sequence derived from the amplicon was highly suggestive of the presence of WNV lineage 2.

Bird migration has been considered one of the major drivers for translocation of WNV (41–43). The emergence of WNV lineage 2 in Hungary followed by dissemination, both locally and to countries to the south could have resulted from translocation through infected bird movements. Two of the regions where the virus has emerged are dominated by wetland areas, namely the Po Delta in north-east Italy and the Aliakmonas Delta in northern Greece (44). Such areas attract migrating birds moving north from Africa and then returning south again from breeding grounds in central Europe. These areas are also associated with abundant populations of *Cx. pipiens* complex mosquitoes. Surveillance in these areas has detected WNV in pools of *Cx. pipiens*, and to a lesser extent *Cx. modestus* (45). This could drive the spread of WNV to indigenous bird species and eventually lead to spill-over infection in humans and horses. This coalescence of events, bird migration, landing periods in wetland areas, and peak vector abundance are needed to stimulate emergence, hence human cases tend to occur in late summer. The repeated emergence of WNV lineage 2 over subsequent summers suggests that over-wintering is occurring, supported mainly by phylogenetic evidence (20), although this is by no means conclusive as local re-introductions could give the same result. If over-wintering is occurring then this would likely be through infected adult females, the primary means of survival of *Culex* mosquitoes from 1 year to the next. The prevalence of virus within the population would gradually increase through the subsequent summer, although always remaining at low levels relative to the total population of mosquitoes, but triggering spill-over infection in humans during late summer.

## Disease Caused by WNV Lineage 2 in Europe

West Nile virus infection is typically asymptomatic. However, a febrile self-limiting illness is reported in around 20% of infected humans and is associated with headaches, myalgia, nausea, vomiting, and chills. A papular rash is reported in some cases, but generally symptoms resolve within 7 days (46–48). In approximately 1% of cases, WNV will enter the central nervous system, infecting neurons and cause neuroinvasive disease (49, 50). Neurological forms are varied and can include encephalitis, meningitis, meningoencephalitis, or acute flaccid paralysis (49). Symptoms are exacerbated by old age and immunosuppression. A follow-up study from the 2010 Greece epidemic reported anorexia, muscle weakness, memory loss, and depression to be the most common sequelae in a group of elderly patients who suffered from WNND. Only 31.8% (7/22) patients recovered fully (48–50).

Equines have been reported to be susceptible to lineage 2 strains, with an increased risk of developing WNND. Clinical signs in horses include ataxia, weakened limbs, paresis, complete paralysis, seizures, chewing, partial blindness, and jaundice/hepatitis (16).

Lineage 2 has been reported with varying mortality across the affected regions. The reasons for this may be linked to the previously characterized threonine 249 to proline (T249P) substitution within the NS3 gene, which was present in WNV isolates responsible for the Greek outbreak but not the Hungarian or Italian isolates (16, 51). However, an Italian strain identical to the Nea Santa-Greece 2010 strain (with T249P) was reported from the Veneto region of Italy, although there has been an absence of human disease as had been observed in Greece (25, 52). The Romanian outbreak of WNV lineage 2 in 2010 had an 8.8% fatality rate with 57 cases of WNND compared to a previous outbreak of WNV lineage 1 in 1996, which had a 4.4% fatality rate.

## Phylogeny of WNV Lineage 2 and Strain Variation

West Nile virus has a single-stranded RNA genome of approximately 11 kb in length. The genome encodes three structural proteins and seven non-structural proteins. A large number of complete WNV genomes have now been sequenced and can be used for phylogenetic comparison with emerging viruses. This has greatly assisted in the investigation of the likely origins of WNV emergence. The 2004 lineage 2 WNV strain was isolated from a wild goshawk (*Accipiter gentiles*) in Hungary, and the genomic sequence of this isolate demonstrated closest homology with a group of southern African strains (53). The introduction of WNV lineage 2 into the wetlands of Hungary could have occurred through migratory birds that had become infected in Africa and remained sufficiently viremic during migration to infect mosquitoes in Europe on arrival (23). However, the goshawk is not considered a migratory bird, suggesting that this African lineage 2 strain must have been transmitted by local mosquitoes prior to detection in 2004.

Genetic characterization of the lineage 2 strain detected in Greece confirmed that it was most closely related to the strain that had previously emerged in Hungary (25). Similarly, strains detected in Serbia in 2012 were most closely related to strains previously identified in Greece, Hungary, and Italy (39). The strains detected in Italy in 2013 show closest homology with lineage 2 strains isolated in Italy in 2011 and Austria in 2008 (27). These observations suggest that the spread of lineage 2 throughout Europe was due to spread of the 2004 Hungarian strain, rather than from separate incursions via migratory birds from Africa. Migratory birds could also have played a role in the spread of lineage 2 WNV in Europe during the southerly migration. During autumn, turtle doves (*Streptopelia turtur*) migrate from breeding areas in central Europe to wintering areas in Africa, and serological studies on individual birds on their arrival in resting areas of Greece suggest that they were exposed to WNV lineage 2 in the area of origin in central Europe, and may constitute a candidate for introduction of lineage 2 strains from central Europe to Greece (37).

**Figure 2:**
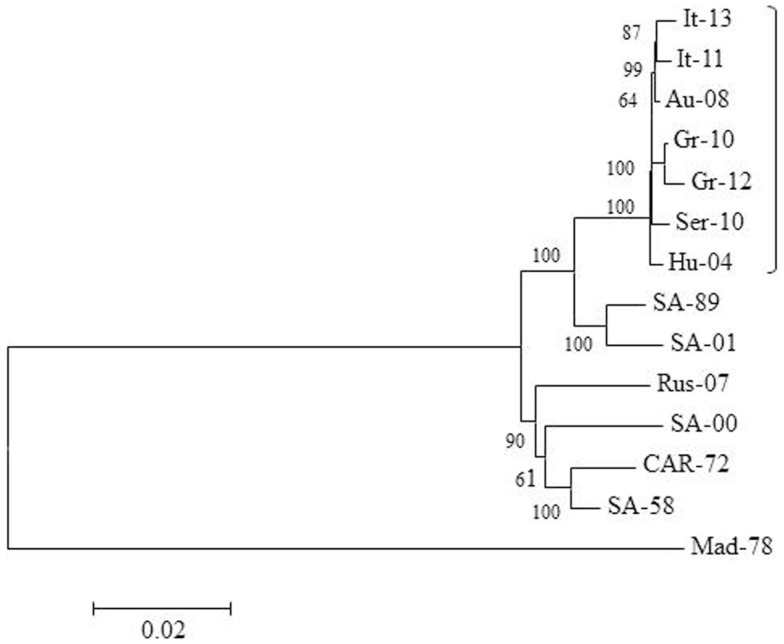
**Phylogeny of WNV lineage 2 in Europe and Africa**. The neighbor-joining tree was generated from an alignment of complete WNV genomes (10,350 base pairs) using MEGA 5 software. Each sequence identifier represents the country of origin and the year of isolation. Further details on each virus are provided in Table 2. Bootstrap values are shown at key nodes and were derived from 1000 replicates. The divergent virus MAD-78 is used as an outgroup.

The mutation T249P within non-structural protein 3 (NS3) of Greek lineage 2 strains is similar to that observed in neuroinvasive lineage 1 isolates (51), while analysis of Italian strains isolated in 2013 has identified a number of amino acid substitutions including E638K and D831G within the NS5 protein (51). Figure 2 highlights the relationship between lineage 2 strains detected in Europe, and the southern African strains from which they may have originated. The isolates used for phylogenetic analysis are detailed in Table 2. The figure demonstrates the close homology between strains from Italy, Austria, Greece, Serbia, and Hungary (marked by a bracket), with clear divergence between these strains and strains from Africa and Russia, suggesting that lineage 2 WNV might now circulate in a separate enzootic cycle within Europe. A number of these viruses were derived from human cases, confirming the zoonotic nature of these outbreaks.

**Table 2:** **Details of WNV lineage 2 sequences used for phylogenetic analysis in Figure 2**.

Sequence ID	GenBank accession no.	Original ID	Species	Country	Year
Hu-04	DQ116961	Goshawk-Hungary/04	*Accipiter gentilis*	Hungary	2004
Gr-10	HQ537483	Nea Santa-Greece 2010	*Culex pipiens*	Greece	2010
Gr-12	KF179639	Greece/2012/Kavala.39.1	Human	Greece	2012
It-13	KF588365	Italy/2013/Rovigo/32.1	Human	Italy	2013
It-11	JN858070	Italy/2011/AN-2	Human	Italy	2011
Au-08	KF179640	Austria/2008-gh	*Accipiter gentilis*	Austria	2008
Ser-10	KC496016	Novi Sad-2010	*Culex pipiens*	Serbia	2010
Rus-07	FJ425721	Reb_VLG_07_H	Human	Russia	2007
SA-89	EF429197	SPU116/89	Human	South Africa	1989
SA-01	EF429198	SA93/01	Human	South Africa	2001
CAR-72	DQ318020	ArB3573/82	*Culex tigripes*	Central African Republic	1972
SA-58	EF429200	H442	Human	South Africa	1958
SA-00	EF429199	SA381/00	Human	South Africa	2000
Mad-78	DQ176636	Madagascar-AnMg798	*Coracopsis vasa*	Madagascar	1978

## Mosquito Species Associated with Disease Transmission: The Case for Emergence in the United Kingdom

Outbreaks of WNV are associated with abundant populations of mosquitoes, which can occur as a result of flooding and subsequent dry and warm weather, or formation of suitable larval breeding habitats (54). Although WNV has been isolated from over 40 species of mosquitoes, the principal mosquitoes involved in WNV transmission belong to the genus *Culex*, particularly species in the *Cx. pipiens* complex (55, 56). Two species are formally recognized in the complex, *Cx. pipiens* (northern, temperate regions) and *Cx. quinquefasciatus* (southern, tropical regions) (57). In the UK, the subgenus *Culex* is represented by the nominotypical *Cx. pipiens* (58), which include two forms, *Cx. pipiens* f. *pipiens* and *Cx. pipiens* f. *molestus*. In addition, *Cx. torrentium* and *Cx. europaeus* are also recorded in the UK (59). These species are fairly common and widespread, with the exception of *Cx. europaeus*, for which few records are available. A further species, *Cx. modestus*, was also believed to be rare in the UK (59). However, recent inventories of UK mosquito fauna have revealed that populations of *Cx. modestus* are well established and commonly found in the North Kent marshes (60, 61).

The occurrence and abundance of potential vector species are a prerequisite for enzootic transmission of mosquito-borne viruses in the UK. Thirty-four species of mosquitoes have been recorded in the British Isles, of which nine species have been implicated in WNV transmission elsewhere (62). Thirteen species are likely to be bridge vectors as they readily bite both birds and humans. In Britain, the ecology of, and the potential risk of WNV transmission by, mosquito species have been detailed by several authors (62–64).

*Cx. pipiens sensu stricto* (*s.s*.) bite both humans and birds; the two forms within this species are morphologically indistinguishable, but they are physiologically different. *Cx. pipiens* f. *pipiens* is mostly ornithophagic and rarely bite humans, the immature stages are found in permanent water, it overwinters in the adult stage and is multivoltine (62). By contrast, *Cx. pipiens* f. *molestus* is highly anthropophilic (though it may bite birds), the immature stages live underground (e.g., flooded basements, sewer tunnels, underground railway systems), and all life stages occur throughout the year (63). The females can be nuisance biters in winter. Medlock et al. (64) stated that the form *molestus* might pose a threat for WNV transmission in suburban and rural areas. Where both forms are sympatric, hybridization can result (65, 66). This leads to increased numbers of mammophilic mosquitoes that can affect transmission of WNV (67). In the UK, the increased use of water containers in private gardens has also been cited as a possible factor that could lead to increases in mosquito abundance in urban areas that in turn could lead to more nuisance biting and increase the risk of WNV maintenance should it be introduced (68).

Golding et al. (60) showed that *Cx. modestus* accounted for 73% of all mosquito species from all sites sampled in the county of Kent and was collected in strong association with *Anopheles maculipennis sensu lato (s.l.)*. Although the risk of WNV transmission to humans in the UK is still low due to limited human exposure to bridge vectors, the risk for transmission was higher in Kent because of the presence of other bridge vector species such as *Cx. pipiens s.l*. and both migratory and resident birds. The authors highlighted the potential risk for horses, which are often grazed in this part of country. At present, screening of specimens of *Cx. modestus* for WNV and other flaviviruses such as Usutu virus is being undertaken by cross-governmental groups in the UK under a “One Health” initiative.

## Invasive Mosquito Species

The increase in human population, expansion of trading routes and tourism, deforestation, and climate change are some of the factors that have facilitated the rapid dispersal of vector species into new geographical areas (69). Recent emergence of bluetongue virus and outbreaks of WN fever and Chikungunya fever in Europe are just a few examples of the risk of exotic vector-borne pathogens being transported to a new region (69).

For invasive mosquitoes in Europe, six exotic species have been identified (*Aedes aegypti, Ae. albopictus, Ae. atropalpus, Ae. japonicus, Ae. koreicus, and Ae. triseriatus*). These have mainly been imported through the international trade in used tires and Lucky bamboo, although public and/or private ground transport have also been implicated. Of these, *Ae. albopictus* presents the greatest threat. Female *Ae. albopictus* lay desiccation-resistant eggs above the surface of the water in tree holes, tires, or other water-holding containers (70). Its ability to breed in artificial containers has facilitated its spread in recent decades along major transportation routes. Currently, *Ae. albopictus* is considered one of the top 100 invasive species of mosquitoes worldwide (71).

*Aedes albopictus* is a known vector of CHIKV, and laboratory infectious studies have shown that this species is a competent vector of WNV (72). It has been recorded in 20 European countries including the Netherlands and Belgium (its northernmost latitude) (73), although it has not been recorded in the UK. Surveillance for this and other invasive mosquito species should be a priority as their presence would radically change the risk of virus emergence.

## Conclusion

West Nile virus lineage 2 was introduced into Europe in 2004 and subsequently emerged in a number of countries. It has overwintered in these countries and in the case of Italy is co-circulating with WNV lineage 1 and Usutu virus. Migratory birds are likely to be the main vehicle of movement for the virus, both as a source of introduction and the means by which it has spread within Europe. However, there is little direct evidence to support this. The disease manifestations appear to vary in different countries. In Hungary, raptors appear to be affected while in Greece the main burden of disease has fallen on the human population. The reasons for this are not clear, but the variation could be caused by mutations within particular virus strains that persist in different locations. The abundance and composition of mosquito populations, particularly *Culex s*pecies, are critical for the spread of disease. Further study of factors such as mosquito distribution, host biting preference, and species hybridization will improve understanding of WNV persistence and assessment of the risk to human populations.

Following detection of WNV, there is a need to implement public health measures to protect at risk populations, particularly the elderly. This includes measures to reduce mosquito biting such as destruction of larval habitats and applications of larvicides. Measures that protect the individual include the application of mosquito repellents and clothing that reduces the exposure of bare skin. Currently, there is no human vaccine available. The risk of further spread of WNV lineage 2 to countries around the Mediterranean Sea is high. Countries in northern Europe appear to be at lower risk due to reduced mosquito abundance and lower winter temperatures. However, constant vigilance is needed to monitor for any change in environmental or ecological conditions that could make the introduction and persistence of WNV in northerly latitudes possible.

## Author Contributions

Nicholas Johnson conceived the idea for this review. Luis M. Hernández-Triana, Claire Jeffries, Karen Mansfield, George Carnell, Anthony Fooks, and Nicholas Johnson prepared the manuscript. All authors read and approved the final manuscript.

## Conflict of Interest Statement

The authors declare that the research was conducted in the absence of any commercial or financial relationships that could be construed as a potential conflict of interest.
